# Rotation Riddle: A Case of Ninety-Degree Rotation of a Plate Haptic Toric Intraocular Lens and Its Management

**DOI:** 10.7759/cureus.97189

**Published:** 2025-11-18

**Authors:** Ishtiaque Anwar, Sharah Rahman, Mehraj Rahman Chowdhury, Nusrat Benta Nizam, Jalal Ahmed

**Affiliations:** 1 Cataract and Refractive Surgery, Bangladesh Eye Hospital and Institute, Dhaka, BGD; 2 Cataract, Cornea and Refractive Surgery, Bangladesh Eye Hospital and Institute, Dhaka, BGD; 3 Ophthalmology, Bangladesh Eye Hospital and Institute, Dhaka, BGD; 4 Cataract and Vitreo-Retina, Bangladesh Eye Hospital and Institute, Dhaka, BGD; 5 Cataract, Refractive Surgery, Glaucoma and Anterior Segment, Bangladesh Eye Hospital and Institute, Dhaka, BGD

**Keywords:** capsular tension ring, high myopia, plate haptic, post-operative rotation of toric iol, toric intraocular lens

## Abstract

The toric intraocular lens (IOL) is the lens of choice for correcting regular corneal astigmatism during cataract surgery. The rotation of the toric IOL frequently encounters challenges, especially in high myopes. In this case report, a patient underwent phacoemulsification surgery with a plate haptic toric IOL implantation to address high myopic astigmatism. However, the patient experienced decreased vision one week postoperatively due to a 90-degree rotation of the toric IOL.

We repositioned the IOL to its new orientation using the Berdahl and Hardten Toric IOL calculator. Additionally, a capsular tension ring (CTR) was inserted. The final visual outcome was satisfactory. IOL re-alignment is a safe and efficient way to recover visual acuity in post-operative toric IOL rotation cases. Toric IOL rotational stability is better ensured with the insertion of a CTR in high myopes.

## Introduction

Cataract surgery is the most common ocular surgery. About 34.8 to 41.3% of cataract patients have astigmatism of one cylindrical diopter (CD) or more [1,2]. Since its introduction in 1992, toric intraocular lenses (IOLs) have gained immense popularity and are now the gold standard in correcting corneal astigmatism. However, residual astigmatism may occur if a toric IOL has the wrong cylinder power or is on the wrong axis. A major reason for axis misalignment is post-operative rotation, of which only 1% have a rotation of 10° or more from the original axis [3]. Such large degrees of rotation may significantly hamper vision, requiring IOL re-rotation. There is also evidence of a positive relationship between the increased axial length of the eye and the rotational instability of toric IOLs post-implantation [4,5]. Below, we report a case of post-operative rotation of a plate haptic toric IOL, about 90° from the original axis in a patient with high myopic astigmatism, one week after cataract surgery and the subsequent management of the case.

## Case presentation

**Figure 1:**
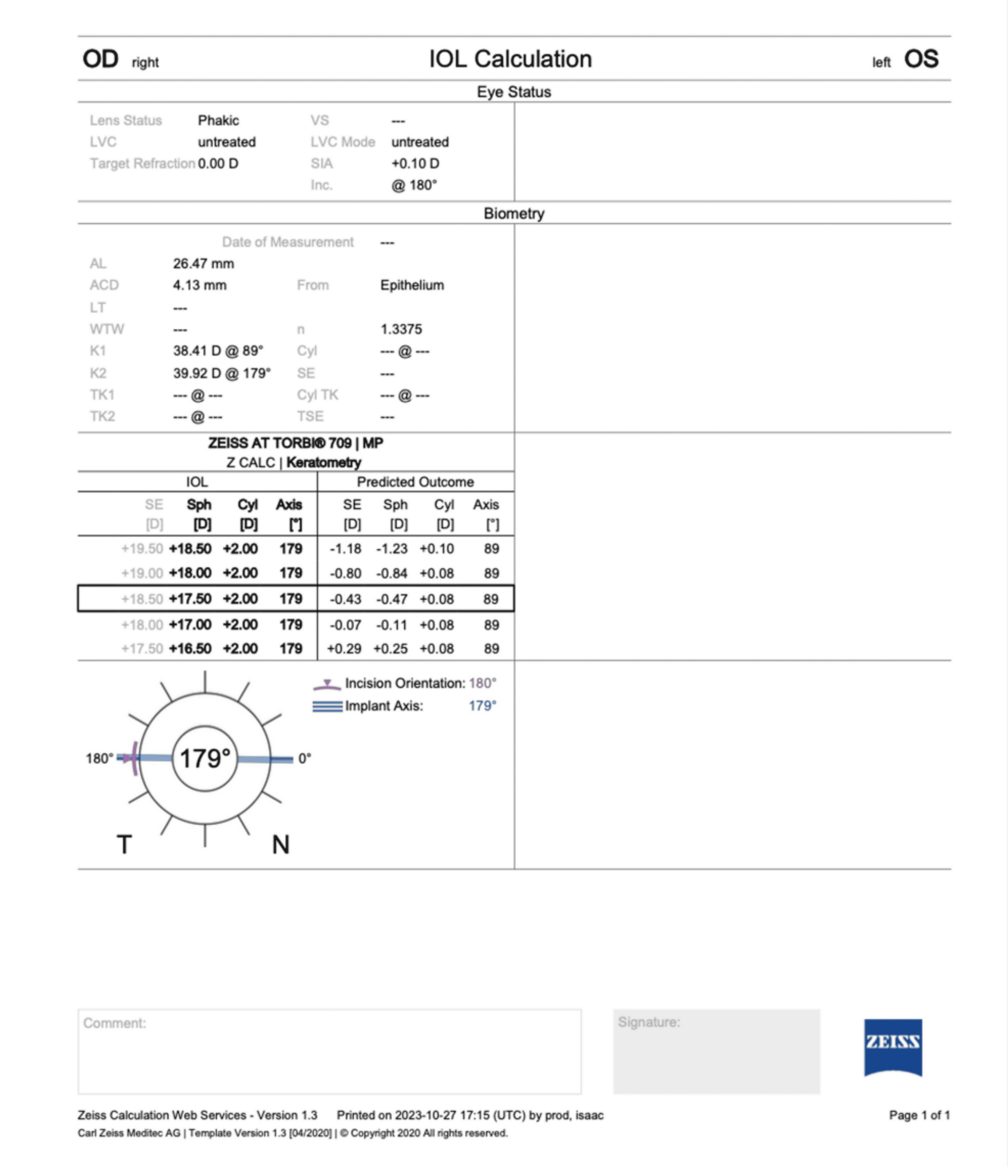
Preoperative biometry using Z CALC online intraocular lens (IOL) calculator https://zcalc.meditec.zeiss.com/ OD: right eye, OS: left eye

A male, 67 years of age, presented with a diminution of vision of the right eye with a best corrected visual acuity (BCVA) of 20/60. Slit lamp biomicroscopy revealed age-related cataracts, and he was advised routine investigations for cataract surgery. Pentacam (OCULUS Optikgeräte GmbH, Wetzlar, Germany) and IOL Master 700 (Carl Zeiss, Oberkochen, Germany) showed that he had astigmatism, which was predominantly corneal and regular. Thus, we planned to implant a toric IOL to correct his high myopic astigmatism. Biometry was performed with IOL Master 700 (Carl Zeiss) and the toric IOL power and axis were calculated with the Z CALC online IOL calculator (https://zcalc.meditec.zeiss.com/) (Figure 1).

**Figure 2:**
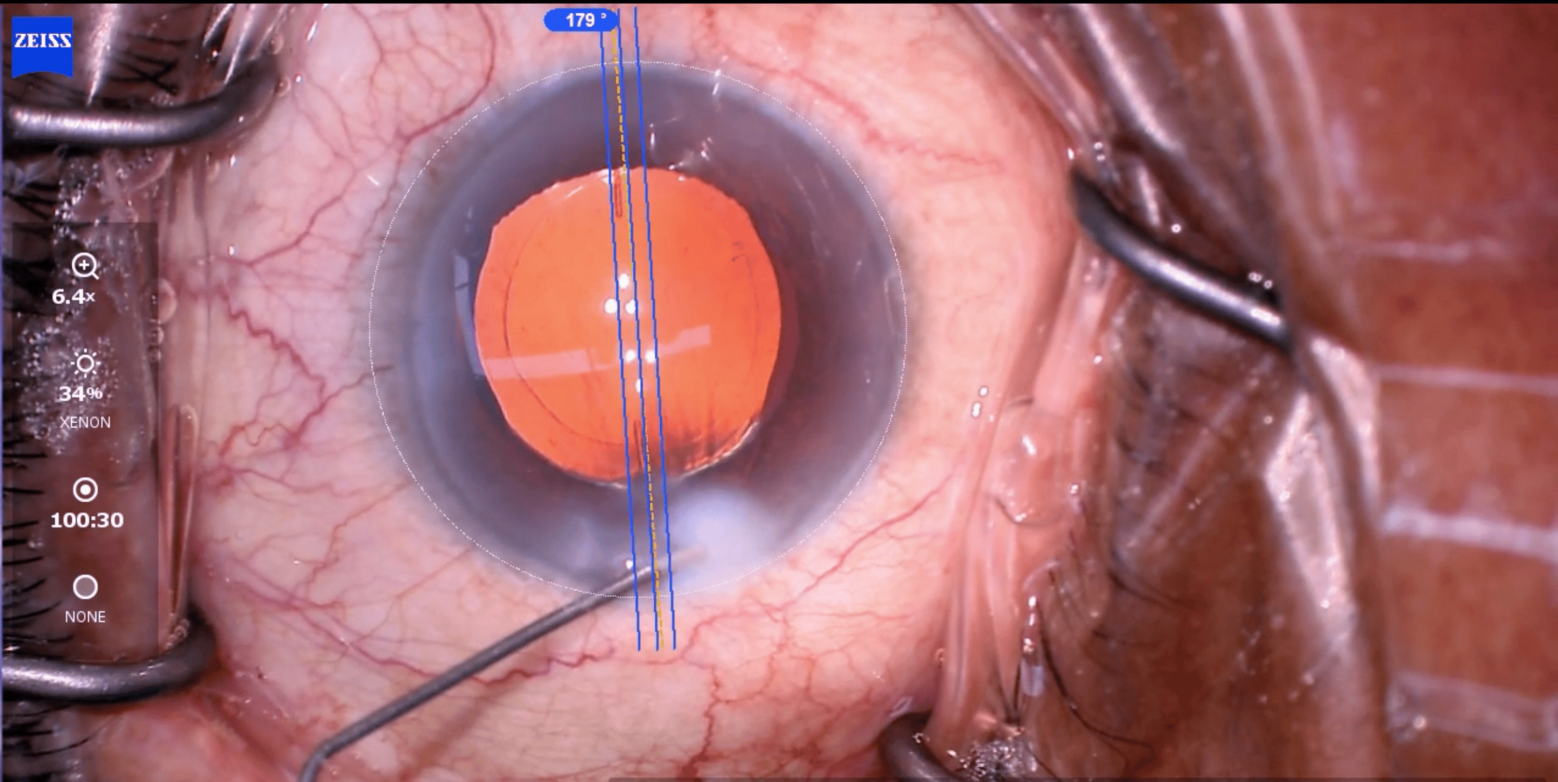
Toric intraocular lens (IOL) axis at the end of primary surgery at 179°

The patient underwent uneventful phacoemulsification surgery with a plate haptic toric IOL implant of power +17.5 spherical diopter (SD), +2.0 cylindrical diopter (CD) at 179° (Figure 2).

Thorough ophthalmic viscoelastic device (OVD) removal was done after IOL implantation from the anterior chamber and under IOL as retained OVD may contribute to post-operative IOL rotation. The chosen toric IOL was an AT TORBI 709MP (Carl Zeiss Meditec AG, Jena, Germany). This is a monofocal, bitoric, aspheric plate haptic IOL. On the first post-operative day, the patient's uncorrected visual acuity (UCVA) was 20/20.

**Figure 3:**
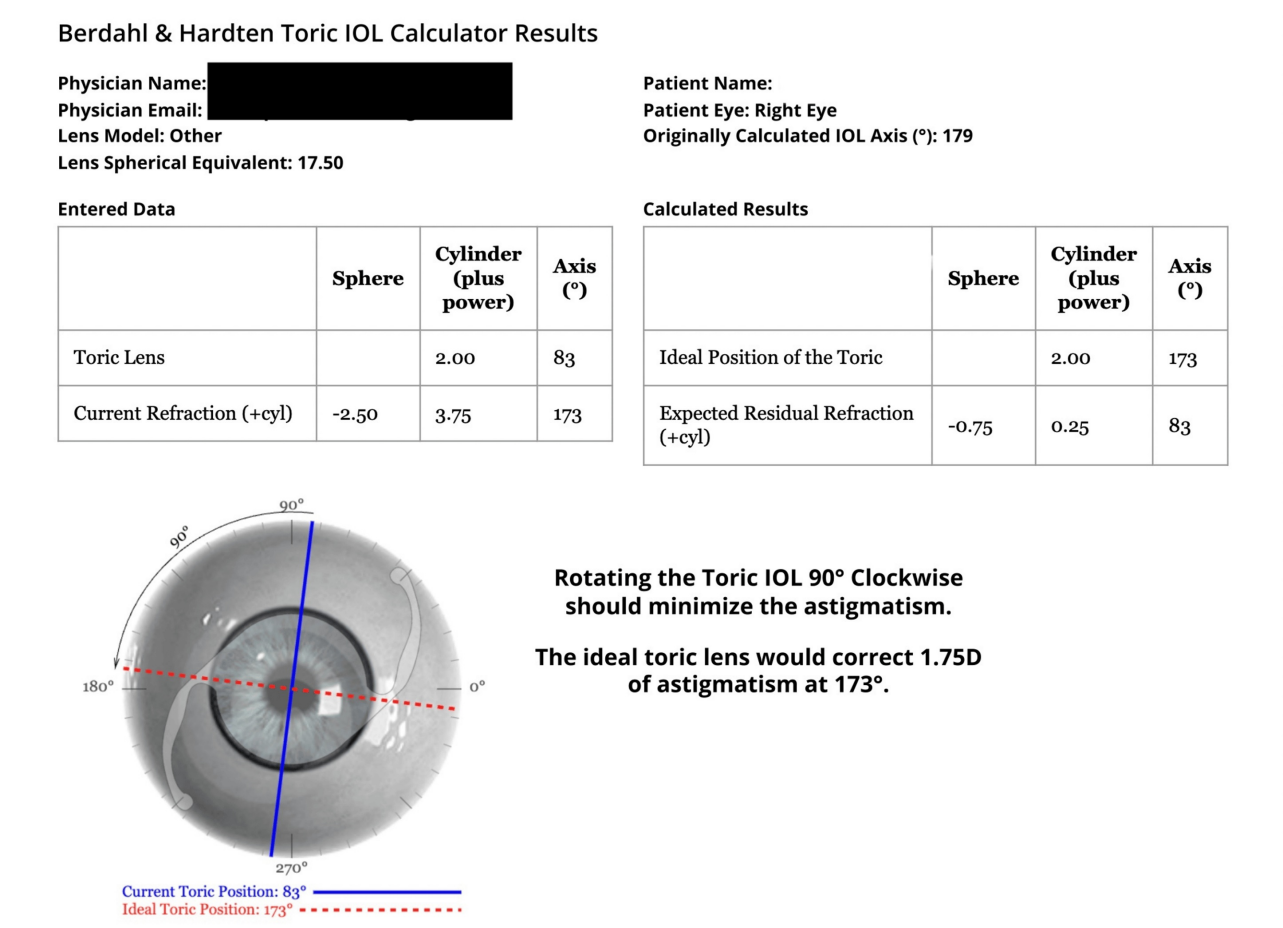
The amount of intraocular lens (IOL) rotation and axis needed was found using the Berdahl and Hardten toric IOL calculator

The patient presented one week after surgery with decreased vision. His UCVA was 20/125, and BCVA was 20/20 with refraction, -2.50 SD, +3.75 CD at 173°. On slit lamp biomicroscopy, we observed the toric IOL had rotated about 90° counter-clockwise from its original position, and we planned to reposition the IOL after further investigations. Berdahl and Hardten's toric IOL calculator was used to calculate the ideal amount of rotation needed and the IOL axis to correct the astigmatism (Figure 3).

**Figure 4:**
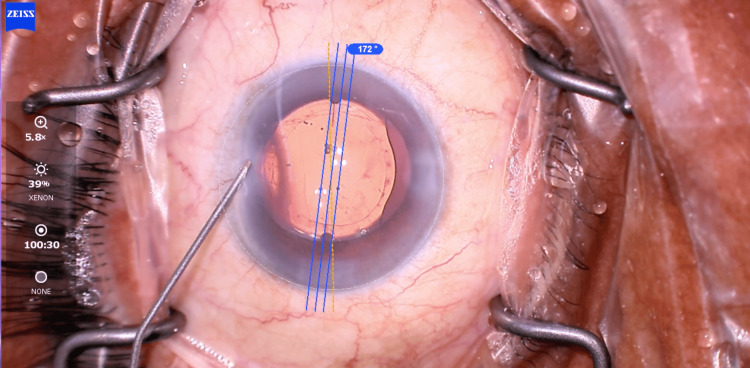
Toric intraocular lens (IOL) axis after re-rotation at 172°

The repositioning surgery was done two weeks after the initial surgery, and the toric IOL was re-rotated from 90° clockwise to 172°. During the procedure, a capsular tension ring (CTR) was also placed inside the capsular bag (Figure 4).

One week after the surgery, the patient had UCVA of 20/20 with auto refractometer reading of -0.50 SD, -0.75 CD at 110° and biomicroscopy showed that the IOL was oriented along the intended axis of placement. After one month, the IOL remained on the correct axis, and the patient's vision was unchanged.

## Discussion

Suboptimal vision may occur with toric IOL implantation when the IOL has the wrong cylinder power or its axis is misaligned. Post-operative rotation is a common complication after toric IOL surgery, especially in high myopes.

Previous studies have shown a definite association between increased axial length of the eye and the rotational instability of toric IOLs post-implantation [4,5]. Our cataract patient had high myopia with an axial length of 26.47 mm. This played a significant role in the large degree of rotation of his toric IOL.

There are different haptic designs from various toric IOL companies, such as loop or plate haptics. Our understanding previously was that post-operative rotation would be less with plate haptic compared to loop haptic IOLs due to the larger contact area of the plates with the fornical area of the capsular bag. However, significant IOL rotation still occurred in our case. Upon further literature review, we found one study comparing the rotational stability of different haptic design toric IOLs, which concluded that the amount of toric IOL rotation was not statistically different between loop and plate haptic IOLs one year after implantation [6].

There is evidence that post-operative misalignment of 10° or more decreases the effectiveness of the toric IOL by 30% [7,8]. In our case, we observed that the toric IOL had rotated about 90° counter-clockwise from its original position, causing significant visual impairment. Re-rotation of the toric IOL was, therefore, an absolute necessity to improve the patient's vision.

High myopes have relatively large capsular bag volumes and increased chances of zonular weakness; as a result, the chance of rotation is higher in these eyes. Implanting a CTR will stretch the fornices of the bag, making it more stable. At the same time, it will bring the posterior capsule into greater contact with the IOL, hence preventing its rotation [9,10]. We followed the advice of these authors and implanted a CTR during the re-rotation surgery. To the best of our knowledge, the use of CTR during the realignment procedure has not been described previously in the literature. Our experience with this case suggests that this may be done in conjunction with IOL re-rotation surgery to prevent it from happening again in the future. One month later, the IOL was still in the correct axis, and the patient was satisfied with UCVA of 20/20.

## Conclusions

Toric IOL rotation is a common complication after cataract surgery, especially in high myopic astigmatism. IOL re-alignment is a safe and efficient way to recover visual acuity in such cases. Toric IOL rotational stability is better ensured with the insertion of a CTR in high myopes. Therefore, the implantation of a CTR may be considered for all high myopic eyes that undergo toric IOL implantation, regardless of haptic design. Long-term follow-up is essential to ensure stable alignment and sustained visual performance.

## References

[1] Ferrer-Blasco T, Montés-Micó R, Peixoto-de-Matos SC, González-Méijome JM, Cerviño A (2009). Prevalence of corneal astigmatism before cataract surgery. J Cataract Refract Surg.

[2] Chen W, Zuo C, Chen C, Su J, Luo L, Congdon N, Liu Y (2013). Prevalence of corneal astigmatism before cataract surgery in Chinese patients. J Cataract Refract Surg.

[3] Ahmed II, Rocha G, Slomovic AR, Climenhaga H, Gohill J, Grégoire A, Ma J (2010). Visual function and patient experience after bilateral implantation of toric intraocular lenses. J Cataract Refract Surg.

[4] Patnaik JL, Kahook MY, Berdahl JP, Hardten DR, Wagner BD, Seibold LK, Kramer BA (2022). Association between axial length and toric intraocular lens rotation according to an online toric back-calculator. Int J Ophthalmol.

[5] Lee BS, Chang DF (2018). Comparison of the rotational stability of two toric intraocular lenses in 1273 consecutive eyes. Ophthalmology.

[6] Miháltz K, Lasta M, Burgmüller M, Vécsei-Marlovits PV, Weingessel B (2018). Comparison of two toric IOLs with different haptic design: optical quality after 1 year. J Ophthalmol.

[7] Felipe A, Artigas JM, Díez-Ajenjo A, García-Domene C, Alcocer P (2011). Residual astigmatism produced by toric intraocular lens rotation. J Cataract Refract Surg.

[8] Berdahl JP, Hardten DR (2012). Residual astigmatism after toric intraocular lens implantation. J Cataract Refract Surg.

[9] Vokrojová M, Havlíčková L, Brožková M, Hlinomazová Z (2020). Effect of capsular tension ring implantation on post-operative rotational stability of a toric intraocular lens. J Refract Surg.

[10] Rastogi A, Khanam S, Goel Y, Thacker P, Kumar P (2018). Comparative evaluation of rotational stability and visual outcome of toric intraocular lenses with and without a capsular tension ring. Indian J Ophthalmol.

